# Amplitude-Integrated/Continuous Electroencephalography for Early Detection of Low Cardiac Output After Chest Closure in an Infant

**DOI:** 10.1016/j.jaccas.2025.105363

**Published:** 2025-09-17

**Authors:** Alessandro Barbaria, Mariarita Capizzi, Federica Sperandeo, Gloria Castelli, Giuseppe Isgrò, Tommaso Aloisio, Angela Satriano, Alessandro Giamberti, Massimo Mastrangelo, Marco Ranucci

**Affiliations:** a Department of Cardiovascular Anesthesia and Intensive Care, I.R.C.C.S. Policlinico San Donato, Milan, Italy; b Department of Infant Neuropsychiatry, ARNAS Civico Di Cristina Benfratelli, Palermo, Italy; c Department of Medical and Surgical Sciences (DIMEC), Alma Mater Studiorum, University of Bologna, Bologna, Italy; d Department of Pathophysiology and Transplantation, University of Milan, Milan, Italy; e Department of Pediatric Intensive Care Unit, I.R.C.C.S. Policlinico San Donato, Milan, Italy; f Department of Pediatric and Adult Congenital Cardiac Surgery, I.R.C.C.S. Policlinico San Donato, Milan, Italy

**Keywords:** acute heart failure, atrial septal defect, congenital heart defect, pediatric surgery, transposition of the great arteries, ventricular septal defect

## Abstract

**Background:**

Weaning from cardiopulmonary bypass in pediatric cardiac surgery is challenging, especially after prolonged procedures. Delayed chest closure may be necessary in cases of low cardiac output syndrome (LCOS) to support hemodynamic recovery. Although near-infrared spectroscopy is standard for neuromonitoring, amplitude-integrated electroencephalography (aEEG) and continuous electroencephalography (cEEG) are emerging tools.

**Case Summary:**

We report the case of a 6-month-old infant with transposition of the great arteries who required delayed chest closure after surgical repair. LCOS recurred after chest closure on postoperative day 3 despite stable near-infrared spectroscopy values. Retrospective aEEG/cEEG analysis, unintentionally initiated before closure, showed early EEG abnormalities preceding clinical signs, which were resolved after chest reopening.

**Discussion:**

This case highlights the potential of aEEG/cEEG to detect early cerebral compromise due to LCOS, even when conventional monitoring appears normal, an association not well established in the literature.

**Take-Home Message:**

Cerebral distress may be detected earlier with aEEG compared with standard monitoring, supporting timely LCOS identification and management.

## Graphical Abstract

**Figure:**
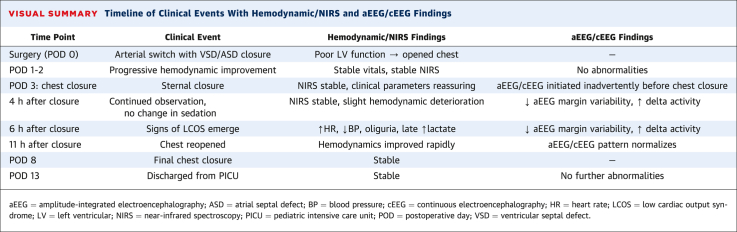

Weaning from cardiopulmonary bypass in pediatric cardiac surgery can be challenging. Prolonged bypass time to repair complex congenital heart disease (CHD) heightens the risk of low cardiac output syndrome (LCOS) owing to myocardial dysfunction, systemic inflammation, and hemodynamic instability. Although standard management includes pharmacological support, fluid optimization, and, in severe cases, extracorporeal life support such as extracorporeal membrane oxygenation,^1^ delayed chest closure is a widely recognized strategy in facilitating successful weaning. Delayed chest closure is particularly beneficial in neonates and infants by mitigating the adverse effects of myocardial edema, diastolic dysfunction, excessive bleeding, and reduced pulmonary compliance.^2^

Accurate assessment of cardiac output, systemic oxygen delivery, and tissue oxygenation is crucial for optimizing hemodynamics and guiding the timing of chest closure, as an increase in intrathoracic pressure may elevate afterload, potentially impairing the cardiac output. In the pediatric intensive care unit, cardiac output is typically assessed using clinical evaluation and hemodynamic parameters, including heart rate, invasive arterial pressure, and right atrial pressure. However, studies have shown that these measures may not reliably reflect true physiological status, highlighting the potential for discrepancies between clinical assessment and actual hemodynamic parameters.^3^ Of note, direct measurement of cardiac output using a pulmonary artery catheter is not feasible in neonates owing to anatomical limitations.

Neonates with CHD have been identified as being at high risk for acute brain injury.^4^ For this reason, neuromonitoring with near-infrared spectroscopy (NIRS) and amplitude-integrated electroencephalogram (aEEG) combined with continuous electroencephalogram (cEEG) has become widely used.^5^ Cerebral NIRS measures regional oxygen saturation, serving as an indirect indicator of oxygen extraction in the underlying brain tissue (typically the frontal cortex). However, NIRS provides only relative measurements, making it most effective for monitoring trends rather than providing absolute values.^6^ Neuromonitoring with aEEG/cEEG has become widely adopted.^7^ The main indication is to assess both the background activity and the burden of electrographic seizures in patients with increased risk of abnormal neurologic outcome, as for example in neonates with CHD.^8^ In particular, aEEG has emerged as a straightforward bedside tool for continuous monitoring of cerebral electrical activity, with interpretations accessible even to nonneurology physicians. A recent prospective observational study^9^ demonstrated that early postoperative EEG background abnormalities in neonates undergoing cardiac surgery strongly correlated with brain lesions on magnetic resonance imaging and predicted adverse neurodevelopmental outcomes. Importantly, EEG patterns were predictive even in the absence of overt clinical signs or significant NIRS abnormalities.

Cerebral electrical activity is closely linked to cerebral oxygen delivery, which depends on optimal cardiac output and sufficient arterial pressure to ensure adequate cerebral blood flow. Of note, although continuous cerebral NIRS assessment is considered the gold standard in the setting of advance hemodynamic monitoring, the role of EEG as a surrogate parameter of cardiac performance is unknown. The aims of this case report are: 1) to demonstrate the occurrence of aEEG/cEEG background abnormalities closely associated with chest closure, with subsequent recovery after chest reopening; 2) to highlight that aEEG/cEEG alterations precede other clinical signs of LOCS; and 3) to explore the potential role of aEEG in the early detection of LCOS.

## History of Presentation

We report the case of a 6-month-old male infant, born full-term via eutocic delivery without significant perinatal complications. At 2 months of age, he developed dyspnea and polypnea during feeding, accompanied by poor weight growth. A cardiologic evaluation was performed, leading to a diagnosis of complex CHD, including transposition of the great arteries, ventricular septal defect, pulmonary stenosis, and pulmonary hypertension. Given these findings, hospitalization was recommended for corrective heart surgery.

## Investigations

On admission, the patient's examination revealed a weight of 6,105 g and a height of 67 cm. Vital signs included a blood pressure of 107/53 mm Hg, heart rate of 150 beats/min, and oxygen saturation of 85% on room air. The electrocardiogram revealed sinus rhythm with a heart rate of 150 beats/min and normal interventricular conduction. Echocardiography (Video 1) revealed situs solitus, levocardia, and atrioventricular concordance with ventriculoarterial discordance; a large atrial septal defect with low-velocity left-to-right shunt, and a large muscular ventricular septal defect with nonrestrictive right ventricle–pulmonary artery flow; a dilated, hypertrophic right ventricle with preserved function and moderate tricuspid regurgitation due to annular dilation; a normal-sized left ventricle with preserved systolic function; unobstructed right ventricular outflow tract, continent tricuspid aortic valve, and normal coronary origins; and mild left ventricle–pulmonary artery flow acceleration and pulmonary regurgitation indicating overflow, with elevated mean pulmonary pressure (58 mm Hg) and dilated pulmonary branches.

## Management

After medical stabilization, the patient underwent surgical repair with arterial switch, atrial septal defect closure, and ventricular septal defect closure with heterologous pericardial patch and patent ductus arteriosus ligation (Video 2).

He was weaned off cardiopulmonary bypass with open chest and inotropic support (adrenaline) because of severe left ventricular disfunction. Monitoring in the pediatric intensive care unit was performed as usual in our center: electrocardiogram, invasive arterial pressure, central venous pressure, NIRS, peripheral oxygen saturation, and urinary catheter. Moreover, advanced neuromonitoring with cEEG (with electrodes placed according to the International 10-20 Reduced System for Neonates) and aEEG was positioned, according to the INNESCO (Italian Neonatal Seizure Collaborative Group) guidelines. After admission, inotropic support was improved with milrinone and levosimendan. Adequate sedation was maintained with continuous infusion of fentanyl and midazolam, and continuous neuromuscular blockade was administered as well. Over the following days, a gradual improvement in hemodynamic status was observed, as indicated by stable vital parameters and consistent NIRS values, accompanied by a reduction in the requirement for pharmacological inotropic support. The improvement led to chest closure on postoperative day 3. Six hours after closing the chest, the patient started developing signs of low cardiac output, with a progressive worsening of hemodynamics (tachycardia, shift from sinus rhythm to junctional rhythm, hypotension) and oliguria-anuria, which led to augmented inotropic support. A late increase in lactates was registered at the arterial blood gas. Notably, there was no modification in cerebral NIRS (Table 1). Eleven hours after chest closure, according to the cardiac surgeon, the chest was reopened, with a consequential improvement in clinical conditions.

**Table 1:** Monitoring Parameters in the Pediatric Intensive Care Unit During Chest Closure and Reopening

	Time												
	8:00	10:00	12:00	14:00	16:00	18:00	20:00	22:00	23:30	24:00	2:00	4:00	6:00
Right cerebral NIRS (%)	79	78	Chest closure	82	78	75	83	77	Chest reopening	81	72	69	72
Left cerebral NIRS (%)	82	83		65	72	67	73	71		95	93	95	92
Internal temperature (°C)	37.3	37.7		37	37	38	39	40.4		39.2	38.4	38	38.1
Rhythm	PM	PM		SR	JR	JR	JR	JR		PM	PM	PM	PM
Heart rate (beats/min)	150	156		163	174	176	184	199		152	154	150	149
Systolic BP (mm Hg)	84	76		69	77	80	86	85		62	76	97	95
Diastolic BP (mm Hg)	45	45		39	41	39	40	40		35	45	54	53
CVP (mm Hg)	11	11		13	13	13	11	11		10	10	10	10
ScvO_2_ (%)	87	86		81	83	72	72	74		63	74	82	80
SpO_2_ (%)	100	100		99	100	93	95	95		100	100	100	100
Urinary output (mL/Kg/h)	1.67	1.25		0.83	2.9	0.83	0.83	0.42		1.5	2.1	1.67	0.83
Lactates (mmol/L)	0.88	—		1.7	1.3	2.9	3	3.65		2.75	2.03	—	—
SvO_2_ (%)	—	—		56	—	—	67	70		—	—	—	—

BP = blood pressure; CVP = central venous pressure; JR = junctional rhythm; PM = pacemaker; NIRS = near-infrared spectroscopy; ScvO_2_ = venous oxygen saturation (PediaSat); SpO_2_ = peripheral oxygen saturation; SR = sinus rhythm; SvO_2_ = venous oxygen saturation.

**Figure 1:**
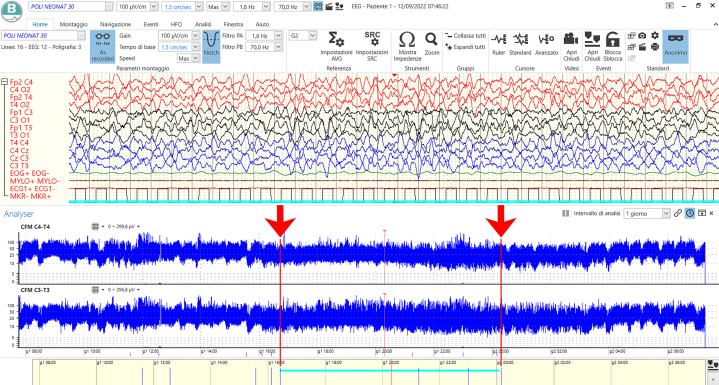
Continuous 23-Hour and 30-Minute Recording of Combined aEEG and cEEG Monitoring in a Patient After Delayed Chest Closure The upper panel displays the raw cEEG signal, while the lower panel shows bilateral aEEG traces (C4-T4 and C3-T3). At 16:24 (left arrow), a clear change in aEEG background is observed, with loss of both upper and lower margin variability, indicative of background suppression. Concurrently, the cEEG shows bilateral high-voltage delta activity (up to 200 μV), most prominent in the anterior regions, with disappearance of faster frequencies. This altered pattern persists until 00:05 (right arrow), suggesting diffuse cerebral dysfunction. The synchronization bar at 20:19:06 (8 hours after chest closure) marks the onset of background slowing on the raw cEEG trace. aEEG = amplitude-integrated electroencephalography; cEEG = continuous electroencephalography.

On the same day, aEEG and cEEG monitoring were inadvertently initiated and continued because of a misunderstanding between the attending physician and the neurophysiology technician, resulting in an unintentional deviation from our protocol, which typically excludes EEG during chest closure for logistical and aseptic reasons; this allowed for the unexpected recording of perioperative cerebral activity. The recording was analyzed and turned out to be particularly significant. At the onset of the recording (Supplemental Figure 1A), the aEEG traces displayed a continuous pattern with good variability in both the lower and upper margins, whereas the cEEG pattern was characterized by continuous theta-delta activity intermingled with superimposed, pharmacologically related fast activity. Four hours after chest closure (Figure 1), aEEG analysis revealed a loss of variability in both margins. Concurrently, the cEEG pattern showed bilateral background slowing (predominantly anterior) associated with high-voltage delta activity and a disappearance of fast activity. This pattern persisted for 9 hours, suggesting diffuse cerebral dysfunction. One hour after sternal reopening (Supplemental Figure 1B), both aEEG and cEEG gradually returned to a reactive and variable baseline pattern. No changes in sedative drug dosing were made during the neuromonitoring period, minimizing pharmacological influence on the observed EEG changes.

## Outcome and Follow-Up

The patient's chest was definitively closed on postoperative day 8. No other significant aEEG/cEEG alterations were registered on subsequent analyses. The patient was dismissed from the pediatric intensive care unit on postoperative day 13.

## Discussion

This case highlights the potential of aEEG combined with cEEG as a sensitive, noninvasive bedside tool for early detection of cerebral compromise secondary to LCOS in infants after complex congenital heart surgery. The use of aEEG/cEEG monitoring in this case (albeit initiated inadvertently) allowed for retrospective analysis and revealed a clear deterioration in cerebral electrical activity, with loss of normal cyclicity and emergence of a low-frequency, high-amplitude delta pattern. These findings resolved promptly upon chest reopening, underscoring the direct relationship between impaired cardiac output and cerebral function. This case report underscores a critical, often under-recognized, vulnerability during the postoperative course of infants recovering from cardiac surgery: The potential for sternal closure to induce acute hemodynamic compromise, even when other parameters appear stable. It also emphasizes the value of multimodal neuromonitoring, particularly the complementary role of aEEG alongside NIRS, in detecting early cerebral distress.

In the presented scenario, aEEG alterations anticipated the clinical and hemodynamic signs of LCOS that developed after delayed chest closure, despite stable cerebral NIRS values. This dissociation suggests that aEEG may detect subtle changes in cerebral function linked to impaired oxygen delivery before they are reflected in more conventional monitoring modalities.

**Visual Summary:** Timeline of Clinical Events With Hemodynamic/NIRS and aEEG/cEEG Findings

Time Point	Clinical Event	Hemodynamic/NIRS Findings	aEEG/cEEG Findings
Surgery (POD 0)	Arterial switch with VSD/ASD closure	Poor LV function → opened chest	—
POD 1-2	Progressive hemodynamic improvement	Stable vitals, stable NIRS	No abnormalities
POD 3: chest closure	Sternal closure	NIRS stable, clinical parameters reassuring	aEEG/cEEG initiated inadvertently before chest closure
4 h after closure	Continued observation, no change in sedation	NIRS stable, slight hemodynamic deterioration	↓ aEEG margin variability, ↑ delta activity
6 h after closure	Signs of LCOS emerge	↑HR, ↓BP, oliguria, late ↑lactate	↓ aEEG margin variability, ↑ delta activity
11 h after closure	Chest reopened	Hemodynamics improved rapidly	aEEG/cEEG pattern normalizes
POD 8	Final chest closure	Stable	—
POD 13	Discharged from PICU	Stable	No further abnormalities

aEEG = amplitude-integrated electroencephalography; ASD = atrial septal defect; BP = blood pressure; cEEG = continuous electroencephalography; HR = heart rate; LCOS = low cardiac output syndrome; LV = left ventricular; NIRS = near-infrared spectroscopy; PICU = pediatric intensive care unit; POD = postoperative day; VSD = ventricular septal defect.

**Equipment List:** 

aEEG/cEEG for early detection of LCOS after chest closure in an infant following cardiac surgery
Device used for newborn/infant EEG video-registration
• BRAIN QUICK® Clinical EEG Video Equipment
Software for aEEG/cEEG analysis
• Software version 1.02.02, “Micromed Group S.p.A.”, Rev. 6.0

## Conclusions

Despite its potential, the role of aEEG as a surrogate marker of cardiac performance remains poorly explored in the current literature, particularly in the context of postoperative LCOS. Incorporating aEEG into standard postoperative monitoring protocols, especially in high-risk patients or during critical transitions such as chest closure, may enhance clinical decision-making and improve outcomes. Further research is warranted to validate the predictive value of aEEG in this context. We are currently developing a prospective observational study to investigate the role of early aEEG/cEEG monitoring in infants undergoing chest closure after cardiac surgery.

## Funding Support and Author Disclosures

The authors have reported that they have no relationships relevant to the contents of this paper to disclose.

**Take-Home Messages**

• Amplitude-integrated EEG can detect early cerebral dysfunction due to low cardiac output before clinical or hemodynamic signs emerge, even when near-infrared spectroscopy values remain unchanged.
• Routine integration of amplitude-integrated EEG into postoperative monitoring may enhance early recognition of low cardiac output syndrome and guide timely interventions in high-risk pediatric cardiac patients.

## Appendix

Video 1 Preoperative Echocardiographic Evaluation A comprehensive preoperative transthoracic echocardiographic assessment in a 6-month-old infant diagnosed with complex congenital heart disease, including transposition of the great arteries, ventricular septal defect, pulmonary stenosis and pulmonary hypertension (PH). The sequence includes 1) Apical Four-Chamber View, 2) Parasternal Long-Axis View, 3) Parasternal Long-Axis with Color Doppler, 4) Parasternal Short-Axis View at VSD Level, 5) Parasternal Short-Axis View at Coronary Level, 6) Suprasternal Notch View.

Video 2 Postoperative Echocardiographic Evaluation A comprehensive postoperative transthoracic echocardiographic assessment in a 6-month-old infant following surgical correction of complex congenital heart disease, including arterial switch, atrial septal defect closure and ventricular septal defect closure with heterologous pericardial patch, patent ductus arteriosus ligation. The sequence includes: 1) Apical Four-Chamber View, 2) Parasternal Long-Axis View, 3) Parasternal Short-Axis View, 4) Pulmonary Artery View.

Supplementary File Supplementary Figure 1 and Equipment List

## References

[1] Varrica A., Cotza M., Rito M.L. (2024). Post cardiotomy extracorporeal membrane oxygenation in pediatric patients: results and neurodevelopmental outcomes. Artif Organs.

[2] Kanakis M., Samanidis G., Kolovou K. (2024). Outcomes of delayed chest closure after congenital heart surgery in neonates. Pediatr Med Chir.

[3] Tibby S.M., Hatherill M., Marsh M.J., Murdoch I.A. (1997). Clinicians' abilities to estimate cardiac index in ventilated children and infants. Arch Dis Child.

[4] Ortinau C.M., Smyser C.D., Arthur L. (2022). Optimizing neurodevelopmental outcomes in neonates with congenital heart disease. Pediatrics.

[5] Pardo A.C., Carrasco M., Wintermark P., Newborn Brain Society, Guidelines and Publications Committee (2025). Neuromonitoring practices for neonates with congenital heart disease: a scoping review. Pediatr Res.

[6] Bronicki R.A., Chang A.C. (2011). Management of the postoperative pediatric cardiac surgical patient. Crit Care Med.

[7] Wusthoff C.J., Numis A.L., Pressler R.M. (2025). The American clinical neurophysiology society guideline on indications for continuous electroencephalography monitoring in neonates. J Clin Neurophysiol.

[8] El-Naggar W.I., Keyzers M., McNamara P.J. (2010). Role of amplitude-integrated electroencephalography in neonates with cardiovascular compromise. J Crit Care.

[9] Lin R., Du N., Feng J. (2023). Perioperative EEG background and discharge abnormalities in children undergoing cardiac surgery: a prospective single-centre observational study. Br J Anaesth.

