# Supplementary material for: Amplitude-Integrated/Continuous Electroencephalography for Early Detection of Low Cardiac Output After Chest Closure in an Infant
Source: JACC Case Rep. 2025 Sep 17;30(32):105363. doi: 10.1016/j.jaccas.2025.105363 (PMC12793864; doi:10.1016/j.jaccas.2025.105363)

Supplemental Figure 1.A. A 23h30' combined amplitude-integrated EEG (aEEG) and continuous EEG (cEEG) recording in a post-operative neonatal patient. From the onset of the recording (07:46) to the first red arrow (16:24), the aEEG traces (C4-T4 and C3-T3) demonstrate a continuous background with good variability in both the lower (10-25 uV) and upper (50-100 uV) margins, consistent with normal cerebral activity. Simultaneously, the cEEG shows continuous theta-delta activity with amplitudes up to 150 uV, interspersed with superimposed pharmacologically related fast activity. The synchronization bar at 09:58:01 (two hours before chest closure) confirms the persistence of a reactive and continuous background.

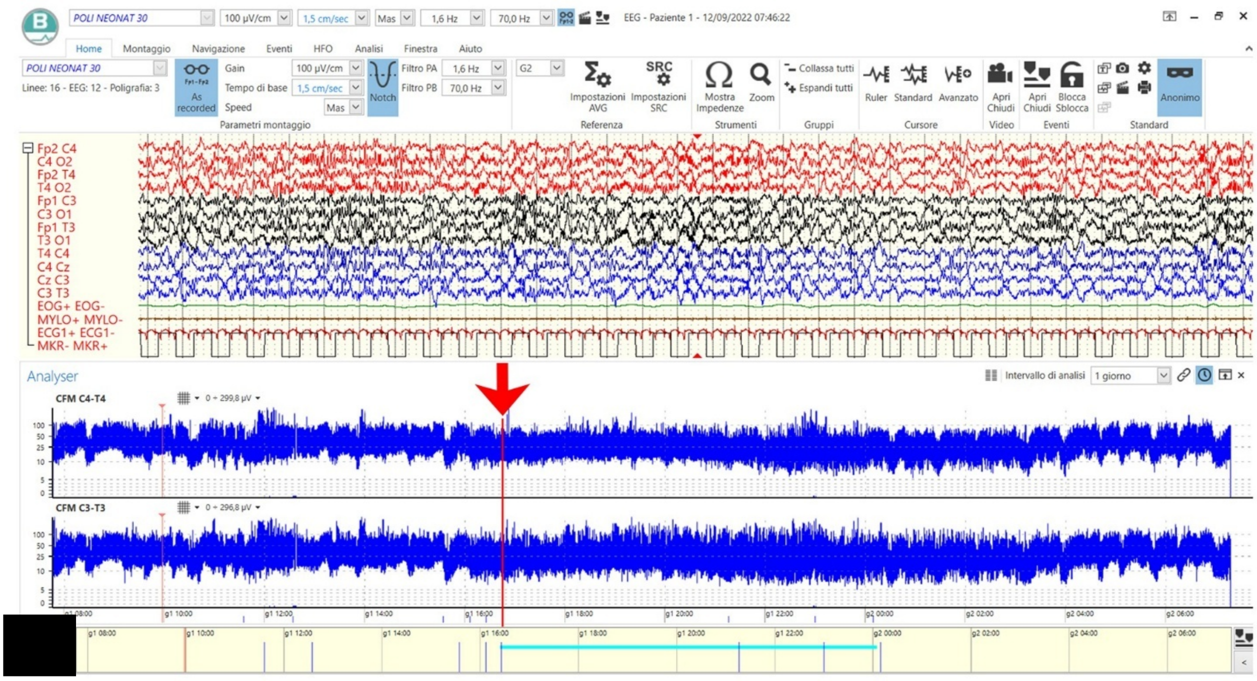

Supplemental Figure 1.B. A 23h30' combined amplitude-integrated EEG (aEEG) and continuous EEG (cEEG) recording in a post-operative neonatal patient. Less than one hour after chest reopening, aEEG traces (C4-T4 and C3-T3) show restored variability of the lower (10-25 uV) and upper (50-100 uV) margins, indicating background recovery. The cEEG displays reappearance of continuous theta-delta activity with superimposed fast frequencies, suggestive of improved cerebral function. The synchronization bar at 06:26:31 confirms the normalization of background activity.

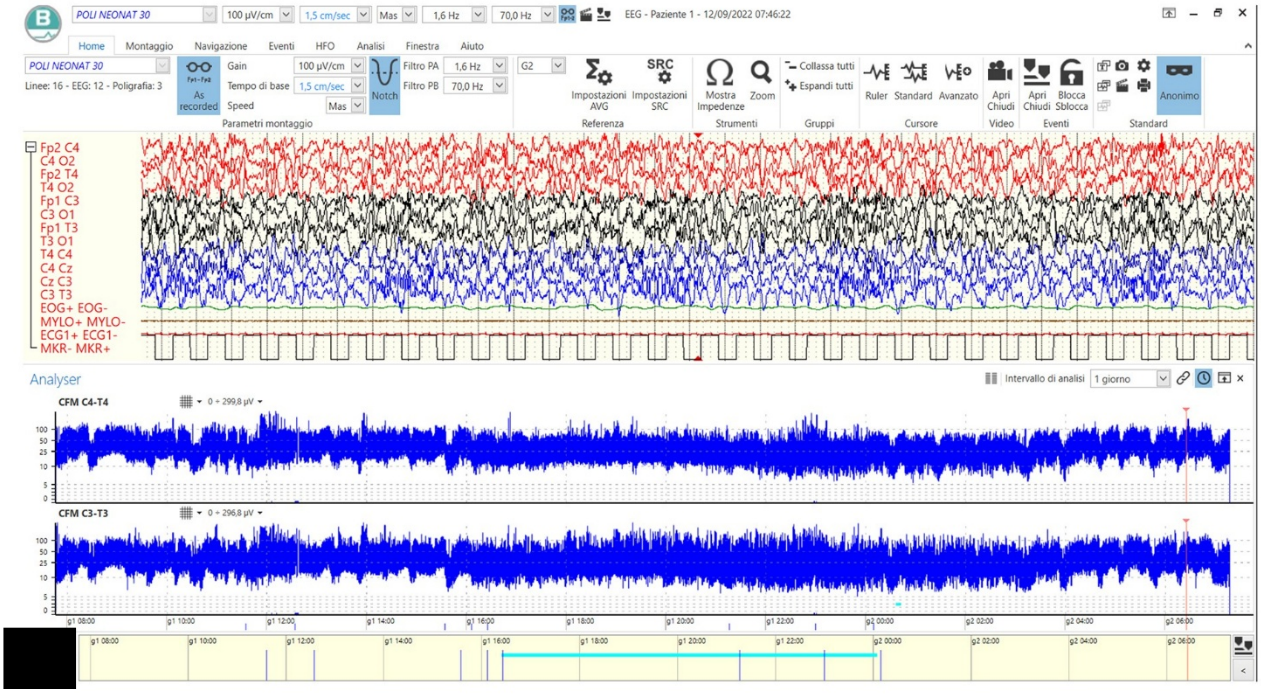

Supplement: Supplementary File — Supplementary Figure 1 and Equipment List [file mmc3.pdf]
